# Identification and Characterization of Novel Antioxidant Protein Hydrolysates from Kiwicha (*Amaranthus caudatus* L.)

**DOI:** 10.3390/antiox10050645

**Published:** 2021-04-22

**Authors:** Sergio Montserrat-de la Paz, Alicia Martinez-Lopez, Alvaro Villanueva-Lazo, Justo Pedroche, Francisco Millan, Maria C. Millan-Linares

**Affiliations:** 1 Department of Medical Biochemistry, Molecular Biology and Immunology, School of Medicine, Universidad de Sevilla, Av. Sanchez Pizjuan s/n, 41009 Seville, Spain; delapaz@us.es (S.M.-d.l.P.); amartinez-ibis@us.es (A.M.-L.); 2 Plant Protein Group, Department of Food and Health, Instituto de la Grasa, CSIC, Ctra. Utrera Km 1, 41013 Seville, Spain; alvarovillanueva@ig.csic.es (A.V.-L.); j.pedroche@csic.es (J.P.); fmillanr@ig.csic.es (F.M.); 3 Cell Biology Unit, Instituto de la Grasa, CSIC, Ctra. Utrera Km 1, 41013 Seville, Spain

**Keywords:** kiwicha, protein hydrolysate, bioactive compound, food ingredient, antioxidant activity

## Abstract

Kiwicha (*Amaranthus caudatus*) is considered one of the few multipurpose pseudocereals for its potential use not only as a source of nutrients and fiber but also for its bioactive compounds. In recent years, antioxidant peptides are commonly used as functional ingredient of food. Herein, a kiwicha protein isolate (KPI), obtained from kiwicha defatted flour (KDF), was hydrolyzed by Bioprotease LA 660, a food-grade endoprotease, under specific conditions. The resulting kiwicha protein hydrolysates (KPHs) were chemically characterized and their digestibility and antioxidant capacity were evaluated by in vitro cell-free experiments owing to their measure of capacity to sequester DPPH free radical and reducing power. KPHs showed higher digestibility and antioxidant capacity than intact proteins into KPI. Therefore, the results shown in this study indicate that KPHs could serve as an adequate source of antioxidant peptides, representing an effective alternative to the generation of functional food.

## 1. Introduction

According to the Food and Agriculture Organization of the United Nations (FAO) we must ensure that food is nutritive and accessible to entire population and that natural resources management preserves the functions of ecosystems to support the satisfaction of current and future human needs. Green industries, such as industrial agricultural emerging crops, offer opportunities for more long-term benefits through sustainable regenerative farming practices. Amaranth, an underutilized crop and a cheap source of proteins, minerals, vitamin A, and vitamin C, seems to be a future crop which can substantiate this demand due to its tremendous yield potential and nutritional qualities, also recently gained worldwide attention [1]. According to the World Health Organization (WHO) and FAO, dietary patterns along with lifestyle habits are important modifiable risk factors in relation to the development of diseases such as chronic noncommunicable diseases (NCDs), major contributor to the global burden of disease, and account for up to 72% of worldwide deaths [1]. Chronic low-grade inflammation, characterized by persistent elevated concentrations of circulating proinflammatory cytokines, across the life span has been associated with the development of both age and diet-related NCDs, including obesity, cardiometabolic diseases, many cancers, respiratory and auto-immune disorders, arthritis, and depression [2]. Persistent inflammation increases the production of reactive oxygen species (ROS) and activates mediators of inflammation as well as suppresses antioxidant defense mechanisms ultimately contributing to oxidative stress which promote development and progression of NCDs [3]. Dietary proteins are a source of bioactive peptides that can exert biological functions and promote health. This current trend towards the use of diet as a global strategy to reduce the incidence and severity of diseases has led to the search for new plant protein sources as an alternative therapy to classical pharmacotherapy.

Amaranths are pseudocereals which head a group of plants with great potentiality to prevent malnutrition in countries with low-income and food-deficit [4]. At the present time, Amaranth is considered one of the few versatile crops which can supply seeds in great quantities that are able to be used in different ways, as tasty leafy vegetables with high nutritional quality, as pseudocereals, and even as food and animal feed [4,5,6]. Grain amaranths are gluten-free compared to others such as wheat, rice and oat [7], and contain 30% more protein and complete set of amino acids [4]. Nowadays, the amaranth seeds are known as kiwicha [8].

The protein composition of amaranths is similar to proteins composition of legumes and crucifers, nevertheless, qualitatively and quantitatively, the protein of their seeds is higher than legumes and cereals [9,10,11,12]. Other pseudocereals such as quinoa (*Chenopodium quinoa* L.), considered as nonconventional sources of protein with excellent nutritional value, also showed a slightly lower protein content in comparison with amaranths grains [13]. Besides the essential role of nutrition in cancer [14], obesity, diabetes, cardiovascular [15], and even in neurodegenerative diseases, which are the leading causes of global deaths, the fact that they include good dietary sources could mitigate or prevent these disorders [1]. Thereby, in the last years, the interest of investigation in bioactive compounds from food has increased as an alternative to pharmacological treatment. The introduction of amaranth seeds into the diet has been linked not only with health promotion but also with prevention of diseases [16].

The main research activities focused on amaranths have been concentrated on their extraordinary nutritive value because of the content of proteins, good fats and bioactive compounds with antihyperlipidemic, antidiabetic [17], antihypercholesterolemic effects [18], and antioxidant [19,20] and antimicrobial activities [21]. Today, amaranth is a traditional food sold mainly as an artisanal product that has been indicated as “functional food”, due to its properties to promote health [22,23,24]. Peptides with low molecular weight (MW) extracted from food proteins can exert different biological properties such as antioxidant, anti-inflammatory, anti-hypertensive, and antibacterial activities. This has resulted in intense research into the likely applications of these kinds of peptides, as those from kiwicha, in the treatment or the prevention of different diseases [8,25].

Oxidative stress appears because of an imbalance between the amounts of produced oxidants and the antioxidant defense mechanisms. Free radicals, such as reactive nitrogen species (RNS) or ROS generated during physiological aerobic metabolism and pathological inflammatory processes, mediate oxidative stress. These ROS and RNS induce damage to DNA as base and nucleotide modifications or strand breaks and oxidation of protein amino acid residues leading to the formation of aggregates by cross-linking, loss of activity in enzymes, disturbance of metabolic pathways or even cell death. Oxidative species can also react with other molecules as low-density lipoprotein (LDL) triggering its oxidation and contributing to increased vascular permeability for example. Thus, oxidative stress contributes to many diseases such as inflammatory and cardiovascular diseases, metabolic syndrome and dementia. Cell membrane integrity can be disrupted by oxidized lipids [26].

Since studies related to antioxidant effect of amaranth-derived peptides are limited, the main objective of this study was to investigate the antioxidant properties of kiwicha protein hydrolysates (KPHs). For that, the specific goals were (i) the isolation of kiwicha protein products and their chemical characterization, (ii) the assessment of the digestibility of KPHs, and (iii) the evaluation of antioxidant effects of the KPHs.

## 2. Materials and Methods

### 2.1. Plant Material and Chemicals

*Amaranthus caudatus* seed flour was provided by STURLA, S.R.L. (Buenos Aires, Argentina). Bioprotease LA-660 was purchased from BIOCON (Barcelona, Spain). All chemicals compounds (reagents and solvents) were provided by Sigma Chemical (St. Louis, MO, USA), Bachem AG, and Gibco, and were of analytical grade and.

### 2.2. Protein Solubility Curve

Kiwicha defatted flour (KDF, 2 g) was dissolved in H_2_O (100 mL) and pH was adjusted to 12 with NaOH. Briefly, pH was taken successively, in 10, 8, 6, 4, and 2 until constant pH, in each point, with HCl. At each pH-point an aliquot was taken, in duplicate, which was centrifuged for 15 min at 12,000 *g*. The supernatants recovered were measured in nitrogen content.

### 2.3. Preparation of Kiwicha Protein Isolate (KPI)

The method of Lqari et al. [27] was used to obtain the KPI. Briefly, KDF was extracted with Na_2_SO_3_ (0.25% *w/v*) for 1 h at pH 11. Then, the extract was centrifugated at 9500 *g* for 15 min. On the one hand, the supernatant was recovered and on the other hand, the pellet was extracted again. The supernatants obtained in both centrifugations were adjusted to the isoelectric point (pI) of kiwicha proteins (pH 4 during 30 min) [12,28]. The precipitate was washed with distilled H_2_O, adjusted to pH 4 and centrifuged in order to remove other non-protein compounds and residual salts. Eventually, the proteins of this precipitate were lyophilized (stored at room temperature).

### 2.4. Hydrolysis of Kiwicha Protein Isolate

KPI hydrolysis was performed under continuous stirring, using a LAMBDA MINIFOR fermenter-bioreactor (Zurich, Switzerland), at controlled conditions of pH and temperature. The KPI was resuspended in distilled H_2_O (10% *w/v*) at 50 °C. Bioprotease LA-660 was added at a ratio enzyme/substrate = 0.3 AU/g protein (pH 8) for 5, 10, 15, 30, and 60 min. To ensure the complete inactivation of remaining enzyme activity each time-point sample was stopped by raising temperature at 85 °C during 20 min. KPH was constituted by the supernatant obtained after centrifugation at 9500 *g* during 15 min. The KPHs obtained at different times of using Bioprotease were designated as 5, 10, 15, 30, and 60, where the number indicates the hydrolysis time in minutes.

### 2.5. Evaluation of Hydrolysis Degree (HD)

The percentage of peptide bonds cleaved is defined as the HD. It was calculated by a method to determine α-amino groups, the 2,4,6-trinitrobenzene sulfonic acid method (TNBS) [29]. A sample that had been hydrolyzed up to 100% in HCl (6N) at 110 °C for 24 h was used as measure of the total number of free amino groups.

### 2.6. Compositional Analysis of Kiwicha Protein Products

The concentration of protein was determined as % nitrogen content × 6.25 by elemental microanalysis using a Leco CHNS932 analyzer (St. Joseph, MI, USA). The gravimetric method was used to determine the total dietary fiber [30]. According to the ignition method (550 °C during 36 h) ash content was determined. Measurement of total phenolic compounds and soluble sugars was undertaken using standard curves of chlorogenic acid for the first and glucose for the second [31,32].

### 2.7. Determination of Amino Acid Composition by Ultra-High-Performance Liquid Chromatography (HPLC)

Amino acid composition was determined according to the method of Alaiz et al. [33] with slight modifications. In this sense, samples were hydrolyzed by incubation in 6 N HCl at 110 °C for 24 h in tubes sealed under nitrogen. Amino acids were determined in the acid hydrolysate by ultra-high-performance liquid chromatography (Acquity Arc, Waters, USA), after derivatization with diethyl ethoxymethylenemalonate, using D,L-α-aminobutyric acid as internal standard, and a 3 mm × 150 mm reversed-phase column (XSelect HSS T3 XP, 2.5 μm; Waters). A binary gradient system with the solvents (A) 25 mM sodium acetate 0.02% sodium azide (pH 6.0), and (B) acetonitrile was used. Calibration curves for each amino acid were developed using a mix of amino acid standard at the same hydrolysis conditions of the samples (Merck, Madrid, Spain) and the resultant peaks were analyzed with EMPOWER software (Waters, Santa Clara, CA, USA). Besides, tryptophan content was assessed according to the Yust et al. method [34].

### 2.8. Pepsin-Pancreactic Digestibility

The method established by Sindayikengera and Xia [35] was slightly modified to determine the protein digestibility in vitro. The sample (0.1 g) was added to a 50 mL tube, with 7.5 mL of HCl 0.1 M containing 1.5 mg of pepsin and then it was incubated at 37 °C for 2 h. Then the suspension was neutralized with 0.5 M NaOH, then treated with pancreatin (1.875 mg) in 7.5 mL of 0.2 M phosphate buffer (pH 8.0). The mixture was gently shaken and incubated for additional 2 h at 37 °C. After this, the sample was treated with 5 mL of 10% TCA (trichloroacetic acid) and centrifuged at 10,000 *g* during 20 min at room temperature. Supernatant proteins were estimated as % nitrogen content × 6.25 by elemental microanalysis in a Leco CHNS932 analyzer. Protein digestibility was calculated as percentage by the ratio of protein in supernatant to total protein in sample.

### 2.9. Molecular Weight (MW) Profile by Fast Protein Liquid Chromatography (FPLC)

MW profiles of protein from the kiwicha products was estimated using FPLC Akta Purifier 10 (GE Healthcare Bio-sciences AB, Switzerland). A prepacked chromatography column for high-performance size exclusion, Superose 12 10/300 GL, with a range for molecules with MWs from 1 to 300 kDa was used. Proteins with MWs known (GE Healthcare, UK) were used to calibrate the column: blue dextran (2000 kDa), aldolase (158 kDa), conalbumin (75 kDa), bovine serum albumin (67 kDa), ribonuclease A (13.7 kDa), and bacitracine (1.423 kDa). Calibration line was made using the logarithms of the MWs of these control proteins and their elution volumes. The elution was accomplished with 50 mL of sodium phosphate buffer (0.05 M), sodium chloride (0.15 M) and sodium azide (0.02% *w/v*) with a flow of 1 mL/min, at pH 7.5. Samples consisted of 500 μL of each one injected at a concentration of 30 mg/mL of protein. Their absorbance at 280 nm was measured to record the elution of the proteins.

### 2.10. Determination of Antioxidant Activity

#### 2.10.1. DPPH Radical-Scavenging Activity

KPI and KPHs effect on scavenging of DPPH free radical was evaluated according to Wu, Chen, and Shiau [36]. In brief, 1.5 mL of each sample with 10 mg/mL of protein was added to 1.5 mL of DPPH (0.1 mmol/L in ethanol). This mix was shaken and kept at room temperature for 30 min. Then, absorbance was measured at 517 nm. Negative control was treated in the same manner using distilled water instead of sample. BHT at 0.08 mg/mL was used as positive standard. Radical DPPH has an absorption band centered at about 517 nm which disappears when an antiradical compound reduces it. Lower absorbance of the reaction mixture means higher DPPH scavenging activity. The scavenging effect was calculated as [(A_control_ − A_sample_)/A_control_] × 100 (A_sample_ and A_control_ are the absorbances respective to sample and control).

#### 2.10.2. Reducing Power

KPI and KPHs ability to reduce iron (III) was assessed according to Oyaizu [37]. Briefly, 0.1 mL of each sample with 10 mg/mL of protein was added to 0.25 mL of sodium phosphate buffer (0.2 M) at pH 6.6 and 0.25 mL of potassiumferricyanide (0.03 M). This mixture was incubated at 50 °C during 20 min. Then 0.25 mL of trichloroacetic acid (0.6 M) was added and it was centrifuged at 1300× *g* for 10 min. In total, 500 µL of supernatant were mixed with 0.5 mL of distilled water and 0.1 mL of ferric chloride (3.7 mmol/L). After 10 min, the resulting solution absorbances were measured at 700 nm. Increased absorbance of the reaction mixture means increased reducing power. As positive standard BHT at 0.08 mg/mL was used.

### 2.11. Statistical Analysis

All values are expressed as arithmetic means ± standard deviations (SD). Graph Pad Prism Version 6.01 software (San Diego, CA, USA) was used to evaluate data. One-way analysis of variance (ANOVA) following Tukey’s multiple comparisons test as post hoc test were used to calculate the statistical significance of differences in each parameter among the groups. Less than 0.05 as *p* value was considered statistically significant.

## 3. Results and Discussion

### 3.1. Chemical Characterization of Kiwicha Protein Products and Protein Digestibility

Kiwicha seed flour was defatted and the resulting KDF presented a protein content of 15.88%, as shown in Table 1. The KPI was extracted from KDF in 30.87% yield by basic extraction and then by acidic precipitation. The resulting KPI showed 84.52% protein content. The chemical composition of KPHs was similar to KPI. These results are similar to those obtained in other pseudocereals of emerging crops as wheat and higher than others as quinoa [38]. The main difference between KPI and KPHs was higher ash content in the hydrolysates because of the addition of alkali to maintain the pH constant during hydrolysis.

According to the Food and Agriculture Organization of the United Nations (FAO) to evaluate of the quality of dietary protein in human nutrition two factors must be considered: absolute and relative quantities of dietary indispensable amino acids and digestibility of protein [39]. In general, most protein from animal sources as eggs, beef, milk, casein and whey, has a higher digestibility than storage proteins of (pseudo)cereals [40]. Protein digestibility is dependent on internal and external factors of them. Some of these external factors are temperature, pH, ionic strength conditions and others as the presence of secondary molecules as antinutritional factors and emulsifiers that limit their digestibility (protease inhibitors as chymotrypsin and trypsin inhibitors), tannins and phytate. Internal factors examples are protein amino acid composition, protein folding and crosslinking. In order to increase the overall digestibility, processing of food is generally designed, affecting these external and internal factors [41]. Removal of protease inhibitors in protein isolate increases the KPI in vitro protein digestibility (77.97 ± 2.93) compared to the KDF (60.74 ± 0.04). The highest value was found for the KPH5, improving digestibility by over 20% with respect to the KDF. Then, the digestibility will decrease slightly, remaining more or less stable during the next 50 min of hydrolysis. This may be because of the generation of new peptides during hydrolysis with Bioprotease, thus, in dietary proteins some specific domains are very stable against digestion and were suggested to be able to cross the mucosal of gut barrier. It has been hypothesized that these peptides would be beneficial to human health, known as bioactive peptides [42]. Despite this, these digestibility values of KPHs are higher than those of other pseudocereals such as buckwheat and legumes such as pigeon pea [43,44].

### 3.2. Amino Acid Composition of Kiwicha Protein Products

The peptide activity might be influenced by amino acid profile in kiwicha products. There are nine essential amino acids, among the 20 types of standard amino acids, that adults cannot produce. The amino acid content in KDF, KPI, and KPHs is reported in Table 2, being the highest concentrations those related to glutamic acid (Glu), aspartic acid (Asp) and arginine (Arg). These profiles have similarities with the amino acid profiles reported in the literature [8]. The importance of these amino acids lies in that Asp is involved in the correct blood circulation, detoxification of toxins in liver and kidneys, and different steps of cellular metabolism and Glu is involved in the neurotransmission, energy transport, and nutrient absorption. Both of them are involved in electron donation mechanisms between ROS. Arg has been involved in nitric oxide synthesis regulation, immune regulation, and insulin, glucagon, growth hormone and prolactin release [26]. There are four essential amino acids identified as limiting amino acids: threonine and lysine in cereals, tryptophan in maize and sulfur amino acids in legumes. Legumes are frequently low in the sulfur-containing amino acids methionine and cysteine, while lysine is typically limiting in grains [45]. The analyses demonstrated that values obtained for kiwicha protein products were far higher than adults requirements established by Food and Agriculture Organization, World Health Organization and United Nations University (FAO/WHO/UNU) for indispensable dietary amino acid [39], reaching double the recommended dose for some of them. The predominant essential amino acids were aromatic amino acids (tyrosine, phenylalanine and tryptophan) and sulfur amino acids (methionine and cysteine), this being relevant since amino acids than contain sulfur represent a powerful part of antioxidant systems of cells [13]. Broadly, no significant differences were observed between protein products, indicating that the enzymatic hydrolysis process is a smooth process and does not harm the various amino acids, nor does it produce secondary metabolites.

### 3.3. Analysis of the Hydrolysis Degree and the Molecular Profile of Kiwicha Protein Products

**Figure 1:**
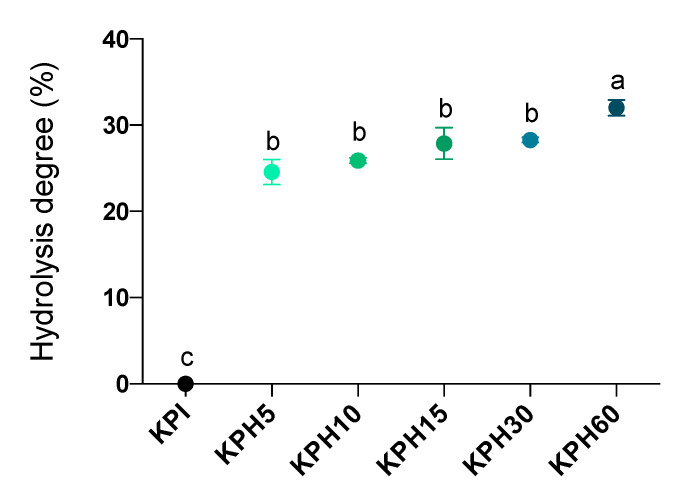
Hydrolysis degree time-course of the KPI during enzymatic hydrolysis by Bioprotease at 5 (KPH5), 10 (KPH10), 15 (KPH15), 30 (KPH30) and 60 (KPH60) min. Data are expressed as the percentage of cleaved peptide bonds (left y-axis). Values are represented as means ± standard deviation of three determinations. Statistical differences are marked with different letters (*p* < 0.05).

The protein hydrolysates include free amino acids, small peptides, and large pep-tides. The extension of hydrolysis can be evaluated by the HD values and it can be used as a measure of the peptide chain length; with higher and lower values indicating shorter and longer lengths, respectively. The extensive hydrolysis of KPI was performed, as said before, using Bioprotease in the way represented in Figure 1. The rate of hydrolysis was faster during the first 5 min and then remained more stable for the following 55 min, reaching a maximum HD of 32.02% (in KPH60). Evaluation of single-stage enzymatic treatments of kiwicha protein using Bioprotease resulted in similar HD values as those obtained by other authors with Alcalase and higher than those obtained with Neutrase and Flavourzyme, all them food-grade enzymes [46].

**Figure 2:**
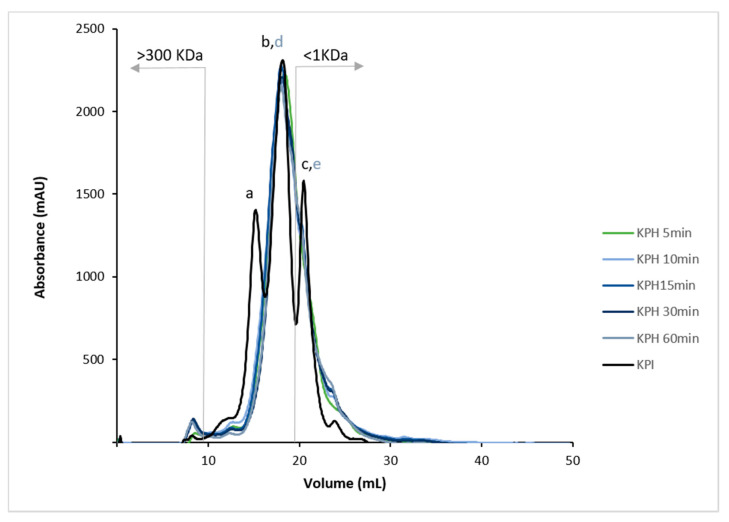
Molecular weight (MW) profiles by FPLC (size-exclusion) chromatogram of KPI and KPH5, KPH10, KPH15, KPH30, and KPH60. MW ranging from 1 to 300 kDa.

The average molecular weight of hydrolysates is a leading factor that determines their biological properties. Reportedly, low-MW peptides, from 2 to 20 amino acids, are more biologically active when compared to their larger parent polypeptide/proteins [47]. As depicted in Figure 2, the protein molecular profile of KPHs showed that the peptide size was around 3.11–0.16 kDa (peaks d and e). This result showed that enzymatic hydrolysis using Bioprotease significantly reduced the protein MW of KPI (15.95 kDa, peak a). Nowadays, most bioactive peptides described have less than 1 kDa. The relationship between the hydrolysates MWs and their bioactivities in human health applications is essential in functional foods. It has been demonstrated that the presence of low-MW peptides enhances the absorption and the ability of amino acids to have bioactive effects [38]. The MW average of kiwicha peptides is lower than MW average of other pseudocereals’ peptides as wheat gluten protein hydrolysates, which after hydrolysis with Alcalase demonstrated to be constituted by peptides with sizes in the range of 1–300 kDa, mainly three of 30.08, 5.36, and 2.19 kDa [38]. In this sense, the smaller size of kiwicha peptides may be attributed to faster hydrolysis with Bioprotease compared to Alcalase. While Bioprotease increased HD up to 20% after 5 min, 10–15 min of hydrolysis with Alcalase are used to reach 20% HD [48].

### 3.4. Determination of the Antioxidant Activity of Kiwicha Protein Products

**Figure 3:**
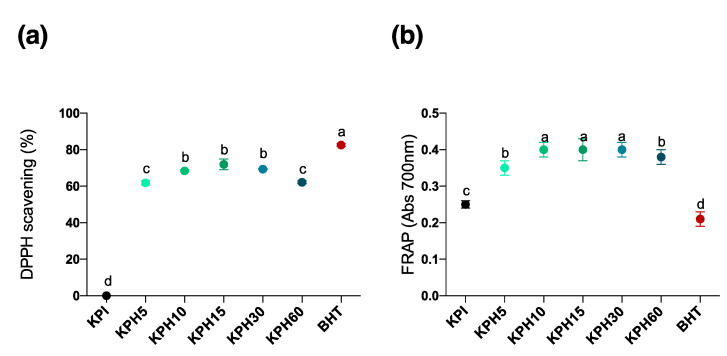
(**a**) DPPH free radical scavenging activity and (**b**) FRAP (ferric reducing antioxidant power) of KPI and KPHs obtained with Bioprotease in an in vitro cell-free experiments. Values are represented as means ± standard deviation of three determinations. Statistical differences are marked with different letters (*p* < 0.05).

Antioxidant peptides are being accepted as food ingredients, as supplement in functional food and nutraceuticals. To further explore the possible antioxidant activity of kiwicha protein products, two assays were carried out: the capacity to sequester DPPH free radical (Figure 3A) and the FRAP (ferric reducing antioxidant power) (Figure 3B). After their enzymatic treatment with different proteases, the antioxidant power of kiwicha products could be enhanced due to the higher exposure of functional amino acid residues in the hydrolysate [49]. BHT was used as positive control in all of the experiments.

Immune response and the control of oxidative stress are two interconnected processes implicated in the maintenance of homeostasis [48]. Inflammation is an important normal immune response during lesions and infections. However, an excessive inflammation can contribute to several acute and chronic diseases characterized by uncontrolled production of proinflammatory cytokines such as tumor necrosis factor-α (TNF-α), interleukin-1β (IL-1) and interleukin-6 (IL-6), eicosanoids derived from arachidonic acid, ROS, and adhesion molecules [50]. Hence, the fine control of the cellular pro-/anti-inflammatory microenvironment is crucial in health maintenance. In this sense, the current trend towards the use of diet as a global strategy to reduce the incidence and severity of diseases has led to the search for new plant protein sources as an alternative therapy to classical pharmacotherapy. Recent studies have shown that bioactive peptides from plant seeds such as lupin or hemp obtained by enzymatic hydrolysis can decrease oxidative stress and inflammatory response in cells of the innate immune system [51,52]. However, the targeting pathways of the immune system by these plant protein hydrolysates are unknown. Most of the food-derived peptides exhibited their anti-inflammatory activities mainly by inhibiting signaling components of either nuclear factor kappa-B (NF-κB) or mitogen-activated protein kinase (MAP)K pathway, which are the two major pathways involved in chronic inflammation following uncontrolled signal activation [53]. Inflammatory pathways impact the pathogenesis of a number of chronic diseases and involve common inflammatory mediators and regulatory pathways. Inflammatory stimuli activate intracellular signaling pathways that then activate production of inflammatory mediators. Inflammatory stimuli, including microbial products such as lipopolysaccharide (LPS), a main constituent of Gram-negative bacterial membrane, and proinflammatory cytokines such as TNF-α, mediate inflammation through interaction with the TLRs or cytokine receptor (TNFR). Receptor activation triggers important intracellular signaling pathways, including the MAPK and NF-κB, whose transcription plays important roles in inflammatory and immune response [54]. Analysis of the antioxidant activity of kiwicha protein products showed that all KPHs enhanced antioxidant activity with respect to the KPI, reaching a maximum DPPH scavenging effect of 77.91% similar to positive control (BHT), and showed much higher levels than BHT reducing power. This antioxidant bioactivity value is similar to that obtained by peptide hydrolysates derived from meat muscle as well as processed meat products like dry-cured ham [55] and better than others from plant sources such as oat, wheat or corn protein [56], which confirmed that identified KPHs may be a good source alternative to animal protein to obtain antioxidant peptides. However, further studies should be performed to elucidate the anti-inflammatory effects of kiwicha peptides.

## 4. Conclusions

Plant protein-based products supplemented in functional food and nutraceuticals are expected to grow considerably in the next years. Plant proteins, usually, present insufficient levels of at least one or even more essential amino acids thus limiting their biological value. However, antioxidant peptides in KPHs presented a balanced amino acid profile and a digestibility higher than intact proteins, hence, these results show that KPHs could be taken into account as an effective option within the functional food generation and would support their use in future clinical food trials.

## Figures and Tables

**Table 1:** Chemical composition of kiwicha protein products.

Fat (Seed Flour, %)			5.73 ± 0.12				
	KDF	KPI	KPH5	KPH10	KPH15	KPH30	KPH60
Proteins	15.88 ± 0.13	84.52 ± 0.55	80.93 ± 0.57	80.77 ± 0.12	80.30 ± 0.36	76.89 ± 0.01	78.50 ± 0.17
Moisture	7.01 ± 0.17	0.96 ± 0.10	2.86 ± 0.13	1.59 ± 0.27	1.29 ± 0.09	4.54 ± 0.04	1.93 ± 0.19
Ash	2.18 ± 0.09	1.75 ± 0.05	7.17 ± 0.25	7.54 ± 0.36	8.40 ± 0.16	9.13 ± 0.59	9.31 ± 0.05
Fiber	10.63 ± 0.16	7.86 ± 0.08	6.58 ± 0.02	7.37 ± 0.09	8.31 ± 0.31	8.09 ± 0.24	8.04 ± 0.18
Sugar	1.73 ± 0.22	0.78 ± 0.12	0.37 ± 0.01	0.52 ± 0.06	0.46 ± 0.02	0.30 ± 0.04	0.29 ± 0.02
Polyphenols	0.01 ± 0.00	0.03 ± 0.00	0.04 ± 0.00	0.05 ± 0.00	0.04 ± 0.00	0.04 ± 0.00	0.04 ± 0.00
Others ^1^	62.56	4.1	2.05	2.16	1.2	1.01	1.89
In vitro protein digestibility ^2^	60.74 ± 0.04	77.97 ± 2.93	79.18 ± 0.66	67.00 ± 3.23	69.58 ± 0.63	71.74 ± 1.66	73.28 ± 0.51

^1^ Others: 100 − (proteins + moisture + ash + fiber + sugar + polyphenols). ^2^ Grams of digested protein/100 g protein.

**Table 2:** Amino acid composition of kiwicha protein products and adult requirements.

AA (%)	KDF	KPI	KPH5	KPH10	KPH15	KPH30	KPH60	FAO ^1,2^
**Indispensable Amino Acids**								
**His**	24.30 ± 0.0	27.60 ± 0.0	30.80 ± 0.1	30.80 ± 0.1	31.20 ± 0.1	31.10 ± 0.0	31.50 ± 0.0	15
**Ile**	30.80 ± 0.3	43.80 ± 0.0	42.70 ± 0.1	44.60 ± 0.1	44.90 ± 0.2	44.90 ± 0.2	45.20 ± 0.1	30
**Leu**	61.20 ± 0.0	72.00 ± 0.1	71.90 ± 0.2	73.90 ± 0.1	74.00 ± 0.1	74.30 ± 0.1	75.60 ± 0.0	59
**Lys**	62.30 ± 0.1	53.10 ± 0.1	55.00 ± 0.1	55.20 ± 0.1	55.00 ± 0.1	55.00 ± 0.0	55.90 ± 0.0	45
**Met + Cys**	32.50 ± 0.1	34.60 ± 0.1	28.20 ± 0.1	35.70 ± 0.1	34.5 ± 0.1	37.10 ± 0.1	36.60 ± 0.1	22
**Met**	13.1 ± 0.1	20.90 ± 0.2	13.3 ± 0.1	21.5 ± 0.1	20.0 ± 0.2	22.7 ± 0.2	20.50 ± 0.2	16
**Cys**	19.40 ± 0.1	13.70 ± 0.0	14.90 ± 0.1	14.20 ± 0.1	14.50 ± 0.1	14.40 ± 0.1	15.10 ± 0.1	6
**Phe + Tyr**	77.90 ± 0.1	92.60 ± 0.1	93.10 ± 0.2	94.80 ± 0.1	93.70 ± 0.1	95.50 ± 0.0	95.40 ± 0.2	38
**Thr**	39.8 ± 0.1	41.0 ± 0.0	42.9 ± 0.1	43.1 ± 0.1	43.0 ± 0.0	43.0 ± 0.0	43.7 ± 0.0	23
**Trp**	9.2 ± 0.0	11.5 ± 0.0	16.7 ± 0.0	16.0 ± 0.0	15.2 ± 0.0	12.6 ± 0.1	8.7 ± 0.0	6
**Val**	35.3 ± 0.3	46.10 ± 0.1	45.2 ± 0.2	47.4 ± 0.1	48.1 ± 0.2	47.30 ± 0.2	48.20 ± 0.2	39
**Total**	373.50	422.30	426.50	441.50	439.60	440.80	440.80	277
**Dispensable Amino Acids**								
**Asp + Asn**	94.3 ± 0.4	92.6 ± 0.2	99.7 ± 0.2	97.7 ± 0.2	97.1 ± 0.1	96.8 ± 0.0	98.3 ± 0.0	n.a
**Glu + Gln**	183.0 ± 0.6	155.1 ± 0.1	168.4 ± 0.3	163.3 ± 0.4	162.4 ± 0.2	160.8 ± 0.0	162.7 ± 0.1	n.a
**Ser**	79.1 ± 0.4	55.4 ± 0.1	60.3 ± 0.1	59.0 ± 0.1	58.5 ± 0.1	58.4 ± 0.1	59.4 ± 0.1	n.a
**Gly**	83.1 ± 0.4	49.3 ± 0.1	52.8 ± 0.1	51.6 ± 0.1	52.5 ± 0.1	51.4 ± 0.0	53.0 ± 0.1	n.a
**Arg**	87.3 ± 0.2	89.4 ± 0.1	95.2 ± 0.2	94.2 ± 0.2	93.8 ± 0.1	93.7 ± 0.1	94.8 ± 0.1	n.a
**Ala**	41.4 ± 0.2	41.1 ± 0.0	43.0 ± 0.1	43.1 ± 0.1	43.3 ± 0.1	43.0 ± 0.0	43.6 ± 0.0	n.a
**Pro**	58.5 ± 0.7	94.9 ± 0.6	54.4 ± 1.9	49.7 ± 1.4	52.7 ± 0.5	54.8 ± 0.6	48.3 ± 1.0	n.a
**Tyr**	34.9 ± 0.1	40.8 ± 0.2	41.0 ± 0.2	41.5 ± 0.1	40.4 ± 0.2	42.3 ± 0.0	41.1 ± 0.3	n.a

^1^ FAO/WHO/UNU. Scoring pattern mg/g protein requirements in adults. ^2^ FAO and FINUT, 2017. Dietary protein quality evaluation in human nutrition. FAO Food and Nutrition Paper NO. 92. n.a: not available.

## Data Availability

Data is contained within the article.
